# Evaluation of ocular psoriasis with meibography^☆^

**DOI:** 10.1016/j.abd.2021.05.008

**Published:** 2021-11-14

**Authors:** Funda Kemeriz, Burcu Tugrul, Erdogan Yasar

**Affiliations:** a Department of Dermatology, Aksaray University Faculty of Medicine, Aksaray, Turkey; b Department of Dermatology, Health Science University Ankara City Hospital, Ankara, Turkey; c Department of Ophthalmology, Aksaray University Faculty of Medicine, Aksaray, Turkey

**Keywords:** Eye diseases, Meibomian gland dysfunction, Psoriasis

## Abstract

**Background:**

Previous studies has shown that dry eye test abnormalities, meibomian gland dysfunction (MGD), may occur in psoriasis.

**Objectives:**

The authors aimed to evaluate the dry eye disease (DED), MGD, in psoriasis patients with meibography which is a current, objective, noninvasive method for patients with meibomian gland diseases, to investigate the relationship between disease severity and ocular involvement.

**Methods:**

This study included 50 participants with psoriasis and 50 healthy individuals. All subjects were examined by the same dermatologist and referred for ophthalmological examination including meibomian gland obstruction, lid margin alterations assessment, ocular surface disease index assessment, tear film break-up time test, Schirmer test, corneal conjunctival fluorescein staining assessment. Additionally, upper and lower lids were evaluated for meibomian gland loss with meibography.

**Results:**

MGD (28%), meibomian gland loss (MGL) (29.5%), upper meiboscore (0.61 ± 0.81), lower meiboscore (0.46 ± 0.61), DED (22%) were significantly higher in the psoriasis group compared with the control group (p = 0.008, p < 0.001, p = 0.027, p = 0.041, p = 0.044, respectively). There was a significant relationship between MGD and psoriasis area severity index (PASI) (p = 0.015, Odds Ratio = 1.211). There was a significant positive relationship between MGL with PASI (p < 0.001, r = 608) and psoriasis duration (p < 0.001, r = 0.547).

**Study limitations:**

Smaller study group and inability to detect quality changes of meibum with meibography were limitations of the study.

**Conclusions:**

Psoriasis may affect the meibomian gland morphology, may cause structural changes in meibomian glands, and as a result of these may cause MGD and DED. Therefore, ophthalmologists and dermatologists should be aware of this situation and co-evaluate the patients in this respect.

## Introduction

Psoriasis is a chronic inflammatory disease that affects 2%–5% of the world population with multiple extracutaneous manifestations.^1^, ^2^ Ocular involvement is particularly common and affects approximately 10% of cases.^3^, ^4^, ^5^ Uveitis, conjunctivitis, blepharitis, and dry eye are associated with psoriasis.^4^, ^6^, ^7^ However, ocular manifestations of psoriasis may be subtle and present subclinically.^6^

The meibomian glands (MG) are sebaceous glands located in the eyelids, and that are responsible for secreting the lipids which play an important role on the ocular surface by preventing the evaporation of tears.^8^

Meibomian gland dysfunction (MGD) is a chronic disease characterized by terminal duct obstruction and/or quantitative-qualitative changes in secretions.^9^ The prevalence of MGD varies between 3.5% and 74.5%.^10^, ^11^ Plugging of MG, Meibomian secretions, telangiectasia, gland loss, as well as a combination of some of these parameters have been used to diagnose MGD.^12^, ^13^ A significant relationship has been found between psoriasis and MGD in studies, but meibography has not been used in the studies with psoriasis although this noninvasive approach has now been widely preferred for clinical use and allowed the undertaking of many clinical studies about meibomian gland diseases.^14^, ^15^, ^16^ In addition, meibography allows objective observation of meibomian glands.^16^

MGD can cause or exacerbate dry eye symptoms, and approximately two-thirds of patients with dry eye disease (DED) have MGD.^17^, ^18^ Thus, it can be said MGD and DED are frequently seen together. Additionally, dry eye test abnormalities have been detected in studies about psoriasis.^19^, ^20^, ^21^

In this study, the authors aimed to evaluate DED and MGD using meibograpy as well as the current diagnostic criteria for patients with psoriasis.

## Materials and methods

### Study design and population

This study was performed with the Institutional Review Board protocol approval, date 19.04.2019 and number 03/59 in Aksaray University Research and Training Hospital, Department of Dermatology and Ophthalmology between May 2019 and August 2019. All study procedures were performed in accordance with the Declaration of Helsinki. Written informed consent was obtained from all participants before the study.

This single-centered, prospective-controlled study included 50 participants (50 eyes) age range from 18 to 65 years, who were clinically and histopathologically diagnosed with psoriasis vulgaris. A parallel (by gender and age) healthy control group (n = 50, 50 eyes) without a family history of psoriasis was also included in the present study.

Participants with ocular infection, allergy, ocular surface disorder, history of ocular surgery or injury, users of topical or systemic drugs which may affect the ocular surface, and users of contact lens were excluded from the present study. Patients who have any dermatologic disease other than plaque-type psoriasis vulgaris and who have systemic diseases and malignancies and patients with Psoralen Ultra-violet A (PUVA) treatment history were not included to the study.

### Diagnosis and assessment of the severity of psoriasis

The disease severity was assessed using Psoriasis Area and Severity Index (PASI). The severity of plaque psoriasis was graded into mild and moderate to severe disease. The mild disease was defined as PASI ≤ 10, and moderate to severe disease was grouped as PASI > 10.^22^ Demographic features and mean PASI scores were recorded and documented.

### Diagnosis and assessment of eye disease

Examinations and tests were performed sequentially as follows: Upper and lower eyelids were assessed with a microscope for obstruction, telangiectasia, irregular lid margins (notching), and a mucocutaneous junction shift. After the examination, fluorescein staining of the ocular surface, Tear Film Breakup Time (TFBUT) testing, Schirmer test, and meibography were performed, and the results of these tests were recorded.

The diagnostic criteria recommended by the MGD Study Group in Japan were used for the MGD diagnosis.^10^ MGD was diagnosed when the MG was occluded, and lid margin abnormalities were present. The BG-4M Non-Contact System was used for meibography (Sirius, Costruzione Strumenti Oftalmici, Firenze, Italy). The MG evaluation was performed with the help of infrared imaging of a slit-lamp biomicroscope and a video camera. The rate of Meibomian Gland Loss (MGL) area to the total area of the glands was calculated by using the software. With this software, the examiner marked the total area and loss of area, and the percentage of the MGL was calculated by the software. MGL was recorded as grade 0 (no loss of MG), grade 1 (0–1/3 of the total MG), grade 2 (1/3–2/3 of the total MG), and grade 3 (>2/3 of the total MG).^23^ Grading of MGL was conducted blindly by the same researcher. MG distortion was recorded as 0 (<50% of the changes) or 1 (>50% of the changes). The meiboscores and MG distortion for the upper-lower eyelids were assessed for the right eye.

The dry eye disease diagnosis was ascertained using the modified Tear Film & Ocular Surface Society Dry Eye Workshop II (TFOS DEWS II) Criteria: OSDI > 13 plus one among TFBUT <10 s, Schirmer test score <10 mm, or corneal and conjunctival staining >0.^24^ TFBUT, Schirmer test, and corneal-conjunctival staining were performed on the right eyes.

### Statistical analysis

The statistical analysis was performed using SPSS version 23.0 for Windows software (SPSS Inc., Chicago, IL, USA). The Shapiro-Wilk test was used to assess whether the distribution of the numerical data was normal. The independent sample *t*-test (for a normal distribution) and the Mann-Whitney test (non-normal distribution) were used to compare the means of numerical variables between the two groups. The Chi-Square test was used to compare the means of categorical variables between the two groups. Spearman’s correlation analysis was used to determine the relationship between the numerical variables that were normally distributed. Binary logistic regression analyses were applied to compute odds ratios for the associations between the explanatory variables; p-values of <0.05 were considered statistically significant.

## Results

Patients diagnosed with psoriasis (n = 50) in this study were 26 (52%) female and 24 (48%) males, and the mean age of 50 patients was 43.4 ± 14.1 (range, 18–65) years. In addition, 25 (50%) of the control group were female and 25 (50%) were male. The mean age of the control group (n = 50) was 41.2 ± 8.1 (range, 21–65) years. There were no statistically significant differences in age and gender between the two groups (p > 0.05).

According to the clinical anamnesis of the patients, 4 of 50 patients (8%) had subjective ocular symptoms, such as itch, burning, and stinging.

The frequency of MGD was 28% (n = 14) in the psoriasis group, and 6% (n = 3) in the control group (p = 0.008). A comparison of Meibomian gland occlusion, telangiectasia, mucocutaneous junction shifts, and lid margin irregularity (notching) is presented in Table 1.

**Table 1:** Comparison of meibomian gland abnormalities between psoriasis and control groups.

	Psoriasis group (n = 50)	Control group (n = 50)	p
Meibomian gland dysfunction, n (%)	14 (28)	3 (6)	0.008
MG occlusion, n (%)	14 (28)	3 (6)	0.008
Telangiectasia, n (%)	27 (54)	12 (24)	0.004
Lid margin irregularity(notching), n (%)	10 (20)	2 (4)	0.031
Mucocutaneous junction shifts, n (%)	11 (22)	3 (6)	0.044
MG loss (%) – Median (min–max)	29.5 (0–69)	9.5 (0–34)	<0.001
Upper Meiboscore – Mean ± SD	0.61 ± 0.81	0.30 ± 0.46	0.027
Lower Meiboscore – Mean ± SD	0.46 ± 0.61	0.24 ± 0.43	0.041
MG distortion, n (%)	9 (18)	7 (14)	>0.05

MG, Meibomian Gland.

MGL was 29.5% in the psoriasis group and 9.5% in the control group (p < 0.001). A comparison of the upper and lower meiboscores is shown in Table 1. No differences in MG distortion were detected between the groups (p > 0.05).

The frequency of DED in the psoriasis group was 22% (n = 11), whereas it was 6% (n = 3) in the control group (p = 0.044). The comparison of the OSDI, TFBUT, Schirmer test, and the corneal staining score is presented in Table 2.

**Table 2:** Ocular surface parameters in the Psoriasis and control groups.

	Psoriasis group (n = 50)	Control group (n = 50)	p
OSDI – Median (min–max)	11.5 (0–23)	7 (0–17)	0.005
Tear film break up time (sec) – Median (min–max)	8 (4–18)	14.5 (6–19)	<0.001
Schirmer test score (mm) – Median (min–max)	14 (5–24)	16 (4–25)	>0.05
Staining score – Mean ± SD	0.52 ± 0.71	0.24 ± 0.62	0.038
Dry eye disease, n (%)	11 (22)	3 (6)	0.044

OSDI, Ocular Surface Disease Index.

Spearman’s correlation test was performed between MGL, PASI scores, and the duration of psoriasis. A significant positive correlation was found between MGL and PASI scores (p < 0.001, r = 0.608) as well as the duration of psoriasis (p < 0.001, r = 0.547).

The relationship between MGD and age, gender, duration of psoriasis, and PASI scores were assessed with a binomial logistic regression test. A significant relationship was found between MGD and PASI scores (p = 0.015, OR = 1.211). No significant relationship was observed between age, gender, and duration of psoriasis (p > 0.05) (Table 3).

**Table 3:** Results of the binomial logistic regression model for psoriasis patients.

	Meibomian gland disease		Dry eye disease	
	p	Odds ratio	p	Odds ratio
Age	>0.05	0.956	0.023	1.073
Gender	>0.05	0.169	>0.05	0.628
Psoriasis duration	>0.05	1.136	>0.05	0,971
PASI	0.015	1.211	>0.05	1.107

PASI, Psoriasis Area and Severity Index.

The relationship between DED and age, gender, duration of psoriasis, and PASI scores were evaluated with a binomial logistic regression test. A significant relationship was found between DED and age (p = 0.023, OR = 1.073). No significant relationship was observed between gender, duration of psoriasis, and PASI scores (p > 0.05) (Table 3).

## Discussion

The data from this present study represent the first report on higher MGL, MGD, and DED in the psoriasis patients than the healthy population with using meibography technique and their positive relationship with disease severity.

In previous studies about psoriasis and MGD which were performed without meibography according to different diagnostic criteria, MGD was found high in patients with psoriasis.^14^, ^15^ Zengin et al. performed a study with 70 psoriasis patients, it was found that patients with psoriasis had higher plugging, thickness indices, and a normal volume of meibomian gland secretion. They claimed that an obstructive type of MGD might result from increased turnover of the epithelia lining the meibomian gland duct in patients with psoriasis.^14^ Aragona et al. performed a study with 66 psoriasis patients, and it was found a significant deterioration of the ocular surface tests, such as tear film lipid layer alteration, tear film instability, corneal and conjunctival epithelial suffering, and mild squamous metaplasia at impression cytology in the patient group compared with the healthy participants.^15^ According to the MGD Study Group in Japan criteria,^10^, ^24^ the frequency of MGD in the present study was significantly higher in 28% of patients with psoriasis, which was consistent with the literature.

Dry eye test abnormalities in patients with psoriasis have been shown in several studies.^19^, ^20^, ^21^ Ghalamkarpour et al. performed a study with 200 psoriatic patients and 100 healthy controls, it was found that the mean values of TBUT and Schirmer's tests in patients were significantly lower than the controls, and significantly higher scores of OSDI were observed among patients compared to the controls. Dry eye disease was observed more frequently in the patients than in the healthy group. They suggested systemic inflammation in patients with psoriasis may play an important role in dry eye abnormalities.^19^ Demirci et al. performed a study with 30 patients with psoriasis and 30 controls, it was found tear osmolarity values, OSDI, Oxford scale scores were significantly higher, and TBUT was significantly lower in the patient group compared with the healthy control group. They suggested psoriasis may effect on tear osmolarity and tear film function by inflammatory processes.^20^ In a study by Santos da Cruz et al. with 43 psoriasis patients and 86 controls, it was found that patients with psoriasis had a statistically higher rate of dry eye (16.28%), likely dry eye (32.56%), blepharitis (16.28%), and ocular surface disease and Rose Bengal tests were more abnormal in patients with psoriasis.^21^ In the present study, according to the TFOS DEWS II criteria,^24^ the frequency of DED was significantly higher in patients with psoriasis (22%), which was consistent with the literature.

The mechanism by which psoriasis induces MGD is not clear. Psoriasis is a chronic inflammatory skin disease characterized by epidermal hyperproliferation and hyperkeratinization.^25^ A large number of cells are produced in this disease, these changes may occur in the Meibomian glands and result in the obstruction of the glands and MGD.^14^, ^15^, ^20^, ^21^ The decreased Meibomian secretions due to MGD in the psoriasis group may have resulted in increased tear evaporation. In addition, DED may have occurred due to increased tear osmolarity from inflammation in patients with psoriasis.^20^ Some similarities exist in the mechanisms of immune privilege of the hair follicle and eye.^26^ Meibomian glands are part of the skin’s sebaceous gland network and are thus responsive to the same inflammatory reactions at work in hair loss. Therefore, having a scalp disease such as psoriasis, lichen planopilaris may occur a risk for MGD and DED.^27^ On the other hand, several studies indicate an association of MGD with inflammatory ocular surface diseases such as Sjögren, conjunctival epithelium could be a direct target of the inflammatory process that leads to the lymphocyte infiltration in the tarsal conjunctiva, and additionally, direct involvement of sebaceous glands occurs in patients with lichen planopilaris^28^, ^29^, ^30^ and, similarly, this involvement may be explained with this mechanism in psoriasis which is a chronic immune-mediated inflammatory disease by T-lymphocytes and dendritic cells.

There are some limitations to this study. First of all, the study included a relatively small number of patients. Secondly, the meibography method only shows morphological changes in the Meibomian glands, so the authors did not determine quality changes in the meibum.

## Conclusion

According to the results of this study, the authors suggest that psoriasis may affect the Meibomian gland morphology, it may cause structural changes in Meibomian glands, and may cause MGD and DED. Meibography is a current objective method, a non-invasive approach which has been widely preferred by clinicians for patients with meibomian gland diseases, and it has allowed many studies about MGD to be carried out. This study is the first report which evaluated MGD in psoriasis patients using meibography technique and their positive correlation with disease severity. On the other hand, the dermatological literature has generally not adequately pointed these complications; however, a thorough understanding and management of ophthalmic involvement are important for the comprehensive care of patients with psoriasis.^6^ Therefore, dermatologists and ophthalmologists should be aware of MGD and DED which may accompany patients with psoriasis. In this study, 4 of 50 patients (8%) had ocular symptoms. Therefore, the authors think that periodic ophthalmological examinations should be performed in patients with psoriasis, especially in those who have high PASI scores, regardless of the presence of ocular symptoms, and early diagnosis of the condition gives patients the chance of treatment, improves the patients’ quality of the life.

## Financial support

None declared.

## Authors' contributions

Funda Kemeriz: Approval of the final version of the manuscript; critical literature review; data collection, analysis and interpretation; effective participation in research orientation; intellectual participation in propaedeutic and/or therapeutic management of studied cases; manuscript critical review; preparation and writing of the manuscript; study conception and planning.

Burcu Tuğrul: Approval of the final version of the manuscript; critical literature review; effective participation in research orientation; manuscript critical review; preparation and writing of the manuscript.

Erdoğan Yaşar: Approval of the final version of the manuscript; critical literature review; data collection, analysis and interpretation; effective participation in research orientation; intellectual participation in propaedeutic and/or therapeutic management of studied cases; manuscript critical review; preparation and writing of the manuscript; statistical analysis; study conception and planning.

## Conflicts of interest

None declared.
